# Confidentiality matters! Adolescent males’ views of primary care in relation to psychosocial health: a structural equation modelling approach

**DOI:** 10.1080/02813432.2022.2144999

**Published:** 2022-12-02

**Authors:** Johanna Haraldsson, Ronnie Pingel, Lena Nordgren, Linus Johnsson, Per Kristiansson, Ylva Tindberg

**Affiliations:** a Department of Public Health and Caring Sciences/Family Medicine and Preventive Medicine, Uppsala University, Uppsala, Sweden; b Centre for Clinical Research Sörmland/Uppsala University, Eskilstuna, Sweden; c Department of Statistics, Uppsala University, Uppsala, Sweden; d Department of Public Health and Caring Sciences/Caring Sciences, Uppsala University, Uppsala, Sweden; e Department of Women’s and Children’s Health, Uppsala University, Uppsala, Sweden

**Keywords:** Adolescent, confidentiality, mental health, risk-taking, latent class analysis, family medicine, consultation process

## Abstract

**Objective:**

To investigate to what degree adolescent males (1) value confidentiality, (2) experience confidentiality and are comfortable asking sensitive questions when visiting a general practitioner (GP), and (3) whether self-reported symptoms of poor mental health and health-compromising behaviours (HCB) affect these states of matters.

**Design:**

Cross-sectional.

**Setting:**

School-based census on life, health and primary care in Region Sörmland, Sweden.

**Subjects:**

2,358 males aged 15–17 years (response rate 84%).

**Main outcome measures:**

The impact of poor mental health and HCBs on adolescent males’ valuing and experiencing private time with the GP, having professional secrecy explained, and being comfortable asking about the body, love and sex, analysed with structural equation modelling.

**Results:**

Almost all respondents valued confidentiality regardless of their mental health or whether they engaged in HCBs: 86% valued spending private time with the GP, and 83% valued receiving a secrecy explanation. Among those who had visited a GP in the past year (*n* = 1,200), 74% had experienced private time and 42% a secrecy explanation. Three-quarters were at least partly comfortable asking sensitive questions. Adolescent males with HCBs were more likely to experience a secrecy explanation (approximative odds ratio [appOR] 1.26; *p* = 0.005) and to be comfortable asking about sex than their peers (appOR 1.22; *p* = 0.007). Respondents reporting experienced confidentiality were more comfortable asking sensitive questions (appOR 1.25–1.54; *p* ≤ 0.010).

**Conclusion:**

Confidentiality matters regardless of poor mental health or HCBs and makes adolescent males more comfortable asking sensitive questions. We suggest that GPs consistently offer private time and explain professional secrecy.

**Key Points**
- Confidentiality for adolescent males has been scantily studied in relation to mental health and health-compromising behaviours.
- In this study, most adolescent males valued confidentiality, regardless of their mental health and health-compromising behaviours.
- Health-compromising behaviours impacted only slightly, and mental health not at all, on experiences of confidentiality in primary care.
- When provided private time and an explanation of professional secrecy, adolescent males were more comfortable asking the GP sensitive questions.

## Introduction

Confidentiality for adolescents in healthcare is recommended by medical organizations worldwide [1,2]. Confidentiality enhances adolescents’ willingness to talk about sensitive topics [3–5], such as symptoms of poor mental health and health-compromising behaviours (HCBs), i.e. behaviours that can impair adolescents’ health or development [6]. Other potentially sensitive topics that many adolescents want to discuss are pubertal development, relationships and sexuality, even though they can be uncomfortable raising such subjects themselves [7,8]. Thus, confidentiality is arguably key to a respectful discussion that includes both health-risk screening and the adolescent’s own concerns.

In this article, *confidentiality* is defined as consisting of two components: *private time* and *secrecy explained*. First, in order to discuss sensitive topics, adolescents need time alone with the healthcare provider, i.e. without guardians being present (*private time*) [1,3,5]. Second, since the meaning and boundaries of professional secrecy can be unclear to adolescents [9], they need an explicit explanation of what professional secrecy entails and when it must be breached (*secrecy explained*).

In Europe, *private time* is reported in 20–35% of adolescents’ medical encounters and *secrecy explained* in 40–67% [10–12]. This indicates missed opportunities to discuss sensitive topics, and important health concerns may thus be overlooked. This is particularly problematic for males 15–19 years old, whose mortality is twice that of their female peers, mostly due to poor mental health and HCBs [6]. Despite higher health risks, adolescent males seek healthcare less often [13], and seem to receive less *private time* and *secrecy explained* than females [4].

Even though confidentiality is pivotal for discussing and intervening against poor mental health and HCBs in adolescent males, studies focusing on confidentiality for adolescent males in relation to mental health and HCBs are scarce and inconclusive. Confidentiality seems to be more important to adolescent males with poor mental health than to those without [14], and HCBs may increase experienced confidentiality, but reports vary [4,15].

General practitioners (GPs) meet many adolescent males with poor mental health or HCBs [13,16–18]. A stable relationship with a GP is associated with less school drop-out [19] and can be an essential part in the treatment of somatic diseases [20]. Adolescent males who have established such a relationship also report less barriers in seeking healthcare [21], and are more likely to consult for poor mental health [22] and to have their anxiety symptoms discussed [23]. To the best of our knowledge, however, it is still unknown whether at-risk adolescent males receive enough confidentiality to actually reveal their problems to the GP. Few studies describe European primary care, and even fewer address whether adolescents are comfortable raising their own concerns. The aim of the study was therefore to investigate to what degree adolescent males (1) value confidentiality, (2) experience confidentiality and are comfortable asking sensitive questions when visiting a GP, and (3) whether self-reported symptoms of poor mental health and HCBs affect these states of matters.

## Methods

### Study design and setting

A cross-sectional study design was used. Data was obtained from the 2014 *Life and Health in Youth*, a triannual, school-based census on health and healthcare. Conducted by the Department of Welfare and Public Health and the Centre for Clinical Research at Region Sörmland, this census targets all schools in the county of Sörmland, Sweden. The Regional Ethical Review Board at Karolinska Institutet, Stockholm, approved the study design (Dnr 2014/1955-32).

### Data collection

In March 2014, school employees distributed questionnaires [24] to the students for completion in their classrooms during ordinary school hours. Students and parents were informed in writing beforehand about the survey and that participation was voluntary. To protect the students’ identities, all parts of the survey were entirely anonymous, and therefore a completed questionnaire was considered as informed consent. Absent students were offered a second opportunity to respond within two weeks. Because questionnaires from both occasions were submitted simultaneously, there is no available information on how many used that opportunity.

### Respondents

Data from males in year 9 of compulsory school (Y9; typically 15 years old), and males in year 2 of upper secondary school (Y2U; typically 17 years old), were used (Table 1). Data from schools for children with intellectual disabilities were excluded.

**Table 1.** Characteristics of the study population.

Characteristics	GP visitors^a^ (*n* = 1,200)	Non-visitors^b^ (*n* = 1,049)	Missing	*p*-value^c^
	n (%)	n (%)	n (%)	
Grade			0 (0.0)	0.305
Y9^d^	562 (46.8)	514 (49.0)		
Y2U^e^	638 (53.2)	535 (51.0)		
Parents’ country of birth^f^:			116 (4.9)	0.031
Sweden	926 (80.8)	843 (83.6)		
Other country in Europe	60 (5.2)	61 (6.1)		
Country outside Europe	160 (14.0)	104 (10.3)		
**Parents’ occupation:**			183 (7.8)	0.927
Both parents work or study	912 (82.2)	812 (82.8)		
One parent unemployed or on sick leave	174 (15.7)	148 (15.1)		
Both parents unemployed or on sick leave	23 (2.1)	21 (2.1)		
**Family situation:**			0 (0.0)	0.805
Living with both parents	704 (58.7)	622 (59.3)		
Living with separated parents	425 (35.4)	360 (34.3)		
Other	71 (5.9)	67 (6.4)		
**Chronic diseases or disabilities:**				
Somatic chronic disease^g^	228 (19.3)	132 (12.8)	41 (1.7)	<0.001
Physical disability^h^	162 (14.0)	93 (9.1)	91 (3.9)	<0.001
Dyslexia	152 (13.7)	120 (12.2)	177 (7.5)	0.312
ADHD^i^ or autism spectrum disorders	113 (10.0)	66 (6.7)	149 (6.3)	0.005

^a^GP visitors: adolescent males who reported that they had visited a general practitioner (GP) once or more often during the past year. The sum of the *GP visitors* and the *Non-visitors* do not equal the total number of respondents (*n* = 2358), due to missing (*n* = 109, 4.6%).

^b^Non-visitors: adolescent males who reported that they had not visited a GP during the past year (including those who did not know).

^c^Difference between adolescent males who had visited a GP during the past year (GP visitors) and those who had not (non-visitors) analysed using Pearson’s chi-squared test.

^d^Y9: year 9 compulsory school.

^e^Y2U: year 2 upper secondary school.

^f^If the parents had different countries of birth, the country geographically closest to Sweden is reported as recommended by Statistics Sweden.

^g^Somatic chronic disease includes asthma, diabetes mellitus, epilepsy, inflammatory bowel disease, and severe allergy. The response options No; Yes, mild; and Yes, severe, were available.

^h^Physical disabilities included are impaired hearing, mobility, or vision (not correctable with glasses).

^i^Attention-Deficit/Hyperactivity Disorder.

### The questionnaire

The questionnaire was composed of 86 questions for Y9, and 87 questions for Y2U. Of these, 80 questions were identical for Y9 and Y2U, and concerned sociodemographic background, somatic and mental health, school situation, relations to parents and peers, HCBs, health promoting behaviours, and confidentiality in primary care. Most questions originated from established questionnaires. Although never validated as a whole, the questionnaire has been previously used in several studies [25–27].

### Variables

#### Outcomes

Four outcome measures were developed for this study. All had good face validity in adolescent males.

*1. Valuing confidentiality (no, yes)*

 Do you value

  a. having the opportunity to speak with the GP in private without your parents?
  b. having the meaning of professional secrecy explained?

*2. GP visitor: Had visited a GP at least once during the past year (no, yes once, yes several times, do not know)*

 Have you visited a GP during the past year?

*3. Experienced confidentiality when visiting a GP (no, yes)*

 When you visited the GP

  c. did you have the opportunity to speak with the GP in private without your parents?
  d. was the meaning of professional secrecy explained to you?

*4. Being comfortable asking sensitive questions when visiting a GP (no, partly, yes)*

 Had you wanted to, would you have been comfortable asking about

   e. your body and appearance?
   f. love and relationships?
   g. sex?

For simplicity, *GP* here denotes any physician working at a primary care centre, because nearly all such physicians in Sweden have completed (or are completing) a residency in family medicine.

#### Exposures

In the analyses, symptoms of poor mental health and HCBs were represented by four latent variables that were developed and validated in data from Swedish adolescent males in Y9 and Y2U through exploratory and confirmatory factor analysis: *unsafety*, *gloominess, pain, and deviancy* (Table 2) [27]. *Unsafety* stands for an inclination to feel unsafe in a large variety of circumstances and locations. *Gloominess* represents a tendency towards poor well-being, pessimism, and not enjoying school, spare time, or life. The third factor, *pain*, describes somatic expressions of poor mental health, including frequently experienced pain in the head, neck, back and stomach. Finally, *deviancy* denotes a tendency to engage in socially deviant behaviours, such as truancy, stealing, and use of tobacco, alcohol, or drugs. Factor analysis groups variables, based on their statistical associations, into validated factors (called latent variables in structural equation modelling, SEM). In the present context, the latent variables represent a continuum from health to risk (details are presented elsewhere [27]). For example, *deviancy* extends from normal healthy experimentation to excessive engagement in multiple HCBs.

**Table 2.** Description of male adolescent GP visitors’ and non-visitors’ behaviours, experiences and symptoms that compose four latent variables and four manifest variables.

The included variables, translated verbatim from the questionnaire	Available response options^a^	GP visitors^b^ *n* = 1200 n (%)^f^	Non-visitors^c^ *n* = 1049 n (%)^f^	Total^d^ *n* = 2358 n (%)^f^	Missing **n (%)**	*p*-value^e^
Have you visited a general practitioner during the past year?	*Yes, once*, *Yes, several times* | No, Do not know	1200 (100.0)	0 (0.0)	1200 (53.4)	109 (4.6)	
***Unsafety***						
Do you feel safe…						
…in the classroom?	Often or always, Sometimes | *Seldom or never*	22 (1.9)	15 (1.5)	38 (1.7)	120 (5.1)	0.448
…in school between classes?	Often or always, Sometimes | *Seldom or never*	24 (2.0)	20 (2.0)	46 (2.0)	102 (4.3)	0.885
…on your way to and from school?	Often or always, Sometimes | *Seldom or never*	22 (1.9)	13 (1.3)	36 (1.6)	83 (3.5)	0.258
…at home?	Often or always, Sometimes | *Seldom or never*	21 (1.8)	10 (1.0)	31 (1.4)	106 (4.5)	0.102
…during daytime, outdoors in your neighbourhood?	Often or always, Sometimes | *Seldom or never*	23 (1.9)	20 (1.9)	45 (2.0)	74 (3.1)	0.977
…during night-time, outdoors in your neighbourhood?	Often or always, Sometimes | *Seldom or never*	39 (3.3)	29 (2.8)	73 (3.2)	82 (3.5)	0.501
*Gloominess*						
Do you enjoy life right now?	A great deal, Quite a bit | *A little bit, Not at all*	97 (8.3)	69 (6.7)	176 (7.8)	101 (4.3)	0.168
How is your general health?	Excellent, Good, Fair | *Poor, Very poor*	38 (3.2)	25 (2.4)	69 (3.0)	30 (1.3)	0.255
During the last three months, how often did you feel cheerful?	*Seldom or never, Once a month, Once a week |* More than once a week, Almost every day	116 (9.9)	91 (8.9)	217 (9.5)	63 (2.7)	0.438
Are you satisfied with your spare time?	Very satisfied, Rather satisfied, Neither satisfied nor unsatisfied | *Rather unsatisfied, Very unsatisfied*	41 (3.5)	35 (3.4)	83 (3.6)	54 (2.3)	0.897
In your spare time, how many times a week do you exercise at least 30 min vigorously enough to make you sweat?	Every day, 4–6 times a week, 2–times a week, Once a wee*k* | *1*–*3 times a month, Less than once a month, Never*	240 (20.5)	272 (26.5)	536 (23.4)	65 (2.8)	0.001
Do you enjoy school?	A great deal, Quite a bit, Somewhat | *A little bit, Not at all*	51 (4.3)	41 (4.0)	100 (4.3)	47 (2.0)	0.659
*Pain*						
During the last three months, how often have you had…						
…headache (not counting migraine)?	Seldom or never, Once a month, Once a week | *More than once a week, Almost every day*	108 (9.4)	70 (7.0)	184 (8.2)	121 (5.1)	0.042
…pain in your neck or shoulders?	Seldom or never, Once a month, Once a week | *More than once a week, Almost every day*	160 (14.1)	99 (10.0)	269 (12.2)	148 (6.3)	0.003
…pain in your back or hips?	Seldom or never, Once a month, Once a week | *More than once a week, Almost every day*	171 (15.1)	91 (9.1)	272 (12.3)	146 (6.2)	<0.001
…stomach ache?	Seldom or never, Once a month, Once a week | *More than once a week, Almost every day*	87 (7.7)	44 (4.4)	135 (6.1)	144 (6.1)	0.002
***Deviancy***						
Do you smoke?	No, Have tried, Have stopped | *Sometimes, Daily,*	206 (17.4)	158 (15.2)	384 (16.5)	37 (1.6)	0.166
During the last 12 months, how often have you been drinking alcohol?	Never, Once, A few times every six month*s* | *1 to 3 times a month, 1*–*2 times a week, More than 2 times a week*	314 (26.7)	221 (21.5)	561 (24.5)	66 (2.8)	0.005
How many times have you ever used illegal drugs?	Never | *Once, Twice or more*	189 (16.1)	133 (12.8)	338 (14.7)	56 (2.4)	0.032
During your last sexual intercourse, did you use any contraception?	Condom, Birth control pill, Contraceptive implant, Other, Emergency contraception, Not yet had their first sexual intercourse | *No contraception was used*	115 (10.1)	83 (8.3)	209 (9.4)	143 (6.1)	0.141
Do you regularly skip class?	No, Once a semester, Once a month, 2–3 times a month| *Once a week, Twice a week or more*	51 (4.3)	49 (4.7)	108 (4.7)	55 (2.3)	0.637
Have you ever…						
…taken goods from a shop without paying?	Never, Once, 2–5 times | *More than 5 times*	110 (9.6)	86 (8.5)	207 (9.3)	131 (5.6)	0.393
…broken into a house or a car?	Never | *Once, 2*–*5 times*, *More than 5 times*	170 (14.9)	101 (10.0)	286 (12.9)	141 (6.0)	0.001
…bought or sold anything you knew, or thought was stolen?	Never | *Once, 2*–*5 times*, *More than 5 times*	148 (13.1)	81 (8.1)	241 (11.0)	163 (6.9)	<0.001
…threatened or forced someone to give you money, a cell phone or similar?	Never | *Once, 2*–*5 times*, *More than 5 times*	50 (4.4)	20 (2.0)	72 (3.2)	140 (5.9)	0.002
…on purpose hit anyone hard enough to cause an injury or bleeding?	Never | *Once, 2*– *times*, *More than 5 times*	376 (32.9)	210 (21.0)	604 (27.3)	147 (6.2)	<0.001
***Impaired connectedness***						
Can you talk about things that you worry about with…						
…your mother?	Yes it is easy, It is neither easy nor difficult | *No it is difficult, I do not see her*	198 (17.0)	176 (17.1)	392 (17.1)	72 (3.1)	0.930
…your father?	Yes it is easy, It is neither easy nor difficult | *No it is difficult, I do not see him*	302 (26.6)	253 (25.0)	575 (25.8)	127 (5.4)	0.418
…other close adult?	Yes it is easy, It is neither easy nor difficult | *No it is difficult, I have no such adult*	383 (35.3)	349 (36.0)	755 (35.3)	218 (9.2)	0.736
Do you have a close friend that you can talk to about everything?	Yes several close friends, Yes one | *None*	159 (13.6)	149 (14.6)	316 (14.0)	104 (4.4)	0.508

Source: Life and Health in Youth 2014.

^a^The responses were dichotomized for readability, with a vertical bar marking the cut-off. Affirmative reports of the response options in italics were summed and are shown as frequencies and proportions.

^b^GP visitors: Adolescent males, who reported that they had visited a general practitioner once or more often during the past year.

^c^Non-visitors: Adolescent males, who reported that they had not visited a general practitioner during the past year (including those who did not know).

^d^Total: Adolescent males from year 9 compulsory school and year 2 upper secondary school, including those who did not answer the question about visiting a general practitioner (*n* = 109).

^e^Pearson’s chi-squared test.

^f^Frequencies and proportions of affirmative responses conforming to any of the italicized options, indicating exposure or negative outcome. Due to missing responses to the question about visiting a general practitioner during the past year (*n* = 109, 4.6%), the sum of *GP visitors* and *Non-visitors* do not equal the n in *Total*.

A confirmatory factor analysis validated the four latent variables among GP visitors in the present study (using the same methods for data generation and estimation as in the study mentioned above [27]). Model fits were acceptable to good (Comparative Fit Index 0.95, Tucker-Lewis Index 0.94, and Root Mean Square Error of Approximation 0.05).

#### Covariates

The included covariates were grade (as a proxy for age) and impaired connectedness, due to their theoretical importance. Age-related differences in health or confidentiality perception are probable, considering the physical, mental, and social development in adolescence. Connectedness to family, school, and peers are among the strongest protecting factors for poor mental health and HCBs in adolescents [28,29]. In the present study, four manifest variables addressed impaired connectedness to parents, supportive adults in school and spare time, and peers: difficulties talking about worries with mother, father, or other close adult, and absence of close friends (Table 2). The most salutogenic response option was coded as zero (talk easily, have several close friends).

### Structural equation modelling

**Figure 1.**
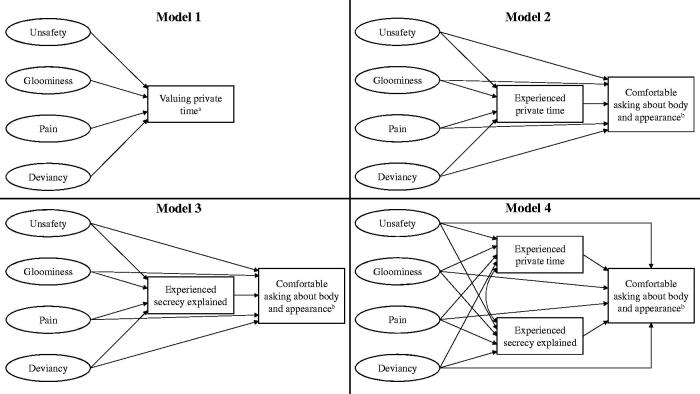
Structural models to study confidentiality and being comfortable asking sensitive questions in relation to mental health (unsafety, gloominess, and pain) and health-compromising behaviours (deviancy). Note that the cross-sectional design limits conclusive evidence regarding the causality in the relationships. Adjustment variables and allowed covariances are shown in Appendix A, Figure A1. ^a^Model 1 was used for seven outcomes: valuing *private time*, valuing *secrecy explained*, experienced *private time*, experienced *secrecy explained*, being comfortable asking about body and appearance, love and relationships, and sex. ^b^Model 2, 3 and 4 were used for three outcomes: being comfortable asking about body and appearance, love and relationships, and sex.

**Figure A1.**
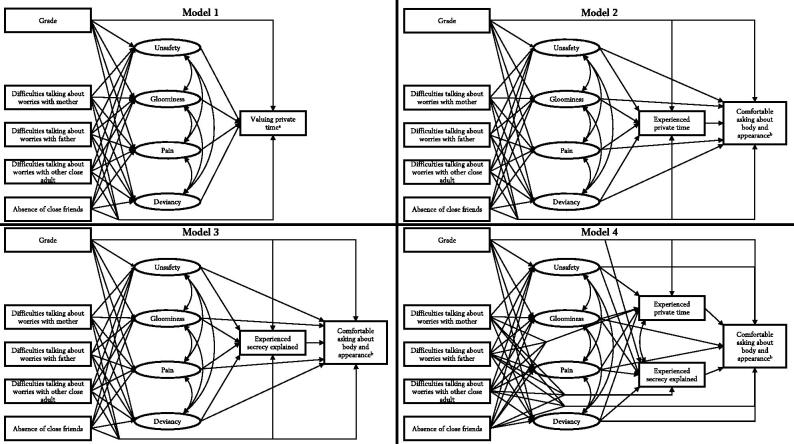
The structural models to study confidentiality and being comfortable asking sensitive questions in relation to poor mental health and health-compromising behaviours, here shown with all included adjustment variables and covariances. Each outcome was tested in relation to poor mental health (unsafety, gloominess, and pain) and health-compromising behaviours (deviancy), adjusted for grade (as a proxy for age) and impaired connectedness (difficulties talking about worries with mother, father, or other close adult, and absence of close friends). Covariances were allowed between the four latent variables (unsafety, gloominess, pain, and deviancy) in all models and between experienced private time and experienced secrecy explained in Model 4. ^a^Model 1 was used for seven outcomes: valuing private time, valuing secrecy explained, experienced private time, experienced secrecy explained, being comfortable asking about body and appearance, being comfortable asking about love and relationships, and being comfortable asking about sex. ^b^Model 2, 3 and 4 were used for three outcomes: being comfortable asking about body and appearance, being comfortable asking about love and relationships, and being comfortable asking about sex.

SEM was used to investigate if self-reported symptoms of poor mental health and HCBs affect whether adolescent males value confidentiality, experience confidentiality, or are comfortable asking sensitive questions when visiting a GP (Figure 1, model 1). Each of these outcomes was analysed in relation to *unsafety*, *gloominess, pain*, and *deviancy*, and adjusted for grade and impaired connectedness (Figure 1, model 1; Appendix A, Figure A1).

Furthermore, because experienced confidentiality might affect being comfortable asking sensitive questions (Figure 1, model 2–4), these three outcomes were adjusted for experienced *private time* (Figure 1, model 2), experienced *secrecy explained* (Figure 1, model 3), and both (Figure 1, model 4) in addition to the variables in model 1. In total, 16 separate SEM analyses were carried out.

### Data analysis

All analyses involving valuing confidentiality were carried out in the total study population (*n* = 2358). Experienced confidentiality and being comfortable asking sensitive questions were only analysable among GP visitors (*n* = 1200). Differences between GP visitors and non-visitors were analysed using Pearson’s chi-squared test (Tables 1–2). A *p*-value <0.05 was considered significant.

Missing data were treated by Multiple Imputation by Chained Equations, creating 20 imputed datasets. SEM analyses were computed using lavaan (sem.mi), PROBIT and theta parameterization. Ordinal data were used and the analyses were adapted for skewed categorical data. The regression coefficients were transformed to approximative odds ratios (appOR) [30].

### Software

R 3.5.1 [31] (package mice [32], lavaan [33] and semTools) in R studio 1.1.463 and Mplus8 (Los Angeles, CA, Muthén & Muthén) [34] were used.

## Results

Of 68 eligible schools, 64 participated with a response rate of 84.5% (85.5% in Y9; 83.6% in Y2U), yielding 2364 completed questionnaires from male respondents (1143 in Y9, 1221 in Y2U). Six questionnaires were considered unreliable and were therefore discarded.

The study population (*n* = 2358) consisted of equal proportions of 15-year-old and 17-year-old adolescent males (Table 1). Most respondents reported good general health, feeling safe in their everyday life, and enjoying school and spare time (Table 2). One-sixth reported smoking regularly, one-fourth consumed alcohol every month, and one-fourth exercised less than weekly.

Half of the respondents (53%) had visited a GP the past year. Compared with non-visitors, GP visitors reported higher frequencies of somatic chronic diseases, Attention-Deficit/Hyperactivity Disorder or autism spectrum disorders, *pain* symptoms, and *deviancy* (Tables 1–2). The only difference in *gloominess* was in the variable about exercise (GP visitors exercised more frequently). GP visitors and non-visitors reported similar frequencies of *unsafety* (Table 2).

### Valuing confidentiality

Of all respondents (*n* = 2358), 86% (*n* = 1865) valued *private time*, and 83% (*n* = 1771) valued *secrecy explained*. Fewer GP visitors than non-visitors valued *private time* (84% versus 88%; *p* = 0.002) or *secrecy explained* (82% versus 86%; *p* = 0.015).

Valuing *private time* was not associated with *unsafety* (appOR 0.89; *p* = 0.257), *gloominess* (appOR 1.16; *p* = 0.123), *pain* (appOR 0.98; *p* = 0.826), or *deviancy* (appOR 1.02; *p* = 0.800), nor was valuing *secrecy explained* associated with *gloominess* (appOR 1.17; *p* = 0.082), *pain* (appOR 1.04; *p* = 0.592), or *deviancy* (appOR 1.11; *p* = 0.163). However, *unsafety* might decrease valuing *secrecy explained* (appOR 0.82; *p* = 0.050). Moreover, compared with Y9, a higher number of adolescent males in Y2U valued *private time* (appOR 1.32; *p* < 0.001) and *secrecy explained* (appOR 1.15; *p* = 0.019). Impaired connectedness had no impact on valuing confidentiality. Altogether, among all respondents, valuing confidentiality was affected by older age, but not by poor mental health or HCBs.

### Experienced confidentiality when visiting a GP

Among GP visitors, 74% (*n* = 837) had experienced *private time* and 42% (*n* = 456) had experienced *secrecy explained*. Of the 924 GP visitors who valued *private time*, 77% had experienced it. Correspondingly, of the 850 GP visitors who valued *secrecy explained*, 47% had experienced it.

*Deviancy* increased the odds of experiencing *secrecy explained* (appOR 1.26; *p* = 0.005; Table 3), and older age increased the odds of experiencing *private time*. None of *unsafety*, *gloomines*s, *pain* or impaired connectedness influenced experienced confidentiality.

**Table 3.** Experienced confidentiality (no, yes) and being comfortable asking sensitive questions (no, partly, yes) in relation to poor mental health and health-compromising behaviours among adolescent males, shown as approximative odds ratios.

	Experienced confidentiality		Comfortable asking sensitive questions											
	Private time (no, yes)	Secrecy explained (no, yes)	Comfortable asking about body and appearance (no, partly, yes)				Comfortable asking about love and relationships (no, partly, yes)				Comfortable asking about sex (no, partly, yes)			
	appOR^a^ *(p-value)*	appOR^a^ *(p-value)*	appOR^a^ *(p-value)*				appOR^a^ *(p-value)*				appOR^a^ *(p-value)*			
Model^b^	Model 1	Model 1	Model 1	Model2	Model 3	Model 4	Model 1	Model2	Model 3	Model 4	Model 1	Model2	Model 3	Model 4
**Confidentiality**														
Experienced private time				**1.68 ** *(<* ** ** *0.001)*		**1.54 ** *(<* ** ** *0.001)*		**1.60 ** *(<* ** ** *0.001)*		**1.43 ** *(<* ** ** *0.001)*		**1.56 ** *(<* ** ** *0.001)*		**1.43 ** *(<* ** ** *0.001)*
Experienced secrecy explained					**1.47 ** *(<* ** ** *0.001)*	**1.25** (*0.010)*			**1.53 ** *(<* ** ** *0.001)*	**1.34** *(0.001)*			**1.43 ** *(<* ** ** *0.001)*	**1.25** *(0.008)*
**Poor mental health**														
Unsafety	1.00 *(0.998)*	0.97 *(0.805)*	0.85 *(0.096)*	0.85 *(0.094)*	0.85 *(0.108)*	0.85 (*0.100)*	0.86 *(0.111)*	0.86 *(0.115)*	0.87 *(0.125)*	0.87 *(0.122)*	0.87 *(0.119)*	0.87 *(0.123)*	0.87 *(0.130)*	0.87 *(0.128)*
Gloominess	1.02 *(0.862)*	0.96 *(0.721)*	1.09 *(0.394)*	1.08 (*0.427)*	1.09 *(0.344)*	1.09 *(0.389)*	1.03 *(0.728)*	1.03 *(0.772)*	1.04 *(0.649)*	1.04 *(0.705)*	1.00 *(0.970)*	0.99 *(0.925)*	1.01 *(0.961)*	1.00 *(0.977)*
Pain	1.03 *(0.755)*	1.03 *(0.722)*	1.16 *(0.062)*	1.15 *(0.078)*	1.15 *(0.071)*	1.15 *(0.080)*	1.13 *(0.118)*	1.12 *(0.139)*	1.12 *(0.135)*	1.12 *(0.144)*	1.11 *(0.161)*	1.10 *(0.184)*	1.10 *(0.183)*	1.10 *(0.192)*
**Health-compromising behaviours**														
Deviancy	1.16 *(0.080)*	**1.26** *(0.005)*	1.04 *(0.594)*	0.99 *(0.933)*	0.99 *(0.869)*	0.97 *(0.699)*	1.16 *(0.050)*	1.11 *(0.160)*	1.09 *(0.231)*	1.08 *(0.307)*	**1.30** *(0.001)*	**1.25** *(0.003)*	**1.24** *(0.004)*	**1.22** *(0.007)*
**Older age**	**1.42** *(<0.001)*	1.13 *(0.069)*	**1.15** *(0.022)*	1.04 *(0.571)*	1.12 *(0.065)*	1.04 *(0.549)*	1.08 *(0.234)*	0.98 *(0.699)*	1.04 *(0.490)*	0.98 *(0.728)*	1.11 *(0.078)*	1.02 *(0.814)*	1.09 *(0.181)*	1.02 *(0.789)*
**Impaired connectedness**														
Difficulties talking about worries with…														
…mother	0.94 *(0.470)*	1.03 *(0.755)*	**0.87** *(0.047)*	0.88 *(0.075)*	**0.86** *(0.038)*	0.88 *(0.061)*	0.88 *(0.077)*	0.90 *(0.121)*	0.88 *(0.061)*	0.89 *(0.091)*	0.88 *(0.070)*	0.90 *(0.113)*	0.88 *(0.062)*	0.89 *(0.095)*
…father	0.94 *(0.469)*	**0.84** *(0.031)*	0.99 *(0.855)*	1.01 *(0.936)*	1.03 *(0.748)*	1.03 *(0.742)*	0.98 *(0.821)*	1.00 *(0.980)*	1.03 *(0.719)*	1.03 *(0.712)*	0.93 *(0.283)*	0.94 *(0.409)*	0.96 *(0.569)*	0.96 *(0.571)*
…other close adult	1.11 *(0.181)*	0.95 *(0.437)*	**0.87** *(0.029)*	**0.84** *(0.007)*	**0.88** *(0.043)*	**0.85** *(0.012)*	**0.88** *(0.049)*	**0.86** *(0.014)*	0.89 *(0.076)*	**0.87** *(0.028)*	0.89 *(0.062)*	**0.87** *(0.021)*	0.90 *(0.091)*	**0.88** *(0.035)*
Absence of close friends	0.94 *(0.430)*	0.94 *(0.342)*	**0.82** *(0.002)*	**0.84** *(0.005)*	**0.84** *(0.005)*	**0.84** *(0.006)*	**0.83** *(0.003)*	**0.84** *(0.006)*	**0.84** *(0.006)*	**0.85** *(0.008)*	**0.83** *(0.002)*	**0.84** *(0.004)*	**0.84** *(0.004)*	**0.85** *(0.005)*

Source: Life and Health in Youth 2014.

^a^Approximative odds ratios derived from regression coefficients in 14 SEM-analyses, each based on 20 imputed datasets (*n* = 1200). Significant associations are highlighted in bold type (*p* < 0.05).

^b^Model 1: Each outcome was tested in relation to poor mental health and health-compromising behaviours, and adjusted for grade (age) and impaired connectedness.

Model 2: Model 1 + experienced private time.

Model 3: Model 1 + experienced secrecy explained.

Model 4: Model 1 + experienced private time and experienced secrecy explained.

### Being comfortable asking sensitive questions when visiting a GP

**Figure 2.**
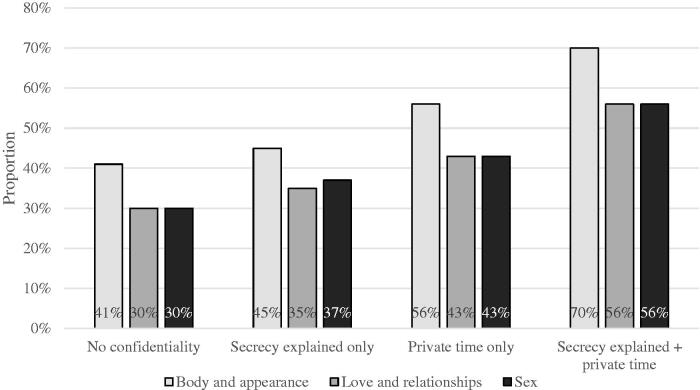
Proportions of adolescent males being comfortable asking about their body and appearance, love and relationships, or sex when visiting a general practitioner in relation to experienced confidentiality: if neither private time, nor secrecy explained were experienced; if secrecy explained was experienced; if private time was experienced; or both. Reports from 1200 Swedish adolescent males (Life and Health in Youth 2014).

Approximately half of the GP visitors would have been comfortable asking sensitive questions and an additional one-fourth reported being *partly* comfortable (asking about their body and appearance: *yes* 57% (*n* = 631), *partly* 22% (*n* = 246); about love and relationships: *yes* 45% (*n* = 492), *partly* 24% (*n* = 263); and about sex: *yes* 45% (*n* = 483), *partly* 24% (*n* = 262)). The proportion increased with experienced confidentiality (Figure 2). Compared with those who reported neither *private time* nor *secrecy explained*, nearly twice as many were comfortable asking sensitive questions among those who experienced both *private time* and *secrecy explained*.

As presented in Table 3, *deviancy* increased the odds of being comfortable asking about sex, but *unsafety*, *gloomines*s, or *pain* did not influence the outcome at all. Difficulties talking about worries with close adults other than parents, and absence of close friends decreased the odds of being comfortable asking sensitive questions. Confidentiality, in particular experienced *private time*, increased the odds of being comfortable asking about body, love and relationships, and sex.

## Discussion

### Statement of principal findings

In the present study, almost all adolescent males valued confidentiality regardless of their mental health or engagement in HCBs. Adolescent males reporting multiple HCBs experienced more frequently *secrecy explained* and were more comfortable asking about sex than peers with fewer HCBs. With these few exceptions, neither symptoms of poor mental health nor HCBs influenced adolescent males’ experienced confidentiality or perception of being comfortable asking sensitive questions. Unsurprisingly, adolescent males were more comfortable asking sensitive questions when *private time* and *secrecy explained* were provided.

### Strengths and weaknesses

The greatest strength of this study is the use of SEM, which takes the well-known clustering of HCBs [35] into account by using a latent variable representing HCBs instead of considering the HCBs one by one [36]. Similarly, the latent variables also enabled use of plenty of poor mental health symptoms instead of psychiatric diagnoses, mirroring the diversity of poor mental health complaints in primary care [13]. SEM also uses a series of regressions in which complex relationships can be analysed simultaneously [36]. However, the cross-sectional design limits conclusive evidence regarding the causality in the relationships [36].

Another strength was the use of self-reported data from an anonymous school-based study with a high response rate, which yielded a large sample and allowed analyses of valuing confidentiality among both GP visitors and non-visitors. Adolescents’ self-reported data of health and healthcare use can be considered reliable [37] and the anonymity is supposed to further improve the data’s accuracy.

Some weaknesses need to be mentioned. First, only associations included in the models are analysed. Important aspects may have been overlooked, and as a result, unmeasured confounders. Another limitation is that the outcomes regarding asking sensitive questions are hypothetical, and may not correctly reflect what really would have happened in the consultation.

### Findings in relation to other studies

#### Valuing confidentiality

Valuing confidentiality was influenced by neither poor mental health nor HCBs. To the best of our knowledge, this association has not been studied before. Given that confidentiality concerns are more common among adolescent males with poor mental health [14], we found the results a bit surprising. It is possible that uncovering such differences requires more detailed questions. However, two covariates stood out. First, in accordance with previous studies, older adolescent males valued confidentiality more than their younger counterparts [12,38], which likely reflects their growing autonomy and increasing interest in discussing sensitive issues with an adult. Second, GP visitors valued confidentiality less than non-visitors, which may indicate differences between GP visitors’ experiences and non-visitors’ expectations. It is possible that many GP-visitors remembered uncomplicated visits, in which confidentiality was not an important issue, whereas the non-visitors visualized other kinds of visits. Another possibility is that non-visitors have refrained from seeking healthcare due to confidentiality concerns as previously described [14]. If true, this would imply that primary care does not deliver appropriate healthcare to all adolescent males, thus jeopardizing their health. Although the present study only regards primary care visits, concerns about lacking confidentiality should be applicable to any healthcare service that fails to explicitly state its commitment to confidentiality.

#### Experienced confidentiality

Three-quarters of the GP visitors reported *private time*, but less than half of them had experienced *secrecy explained*. This may be deeply problematic. Given that many adolescent males are unfamiliar with the limits of confidentiality [9], *private time* without *secrecy explained* can result in broken trust, e.g. if adolescent males come to disclose, unaware of the consequences, matters that require breaching confidentiality. Such experiences may negatively affect future healthcare seeking, compromising their health [1,3,9].

Adolescent males with HCBs more often experienced *secrecy explained*, but no such effect was seen for those with symptoms of poor mental health. This finding contrasts with a study in American primary care, in which *secrecy explained* had a weak association with depressive symptoms, but was unrelated to substance use or sexual activity [15]. Conversely, in another study from the U.S., the regular health-care provider more often explained the professional secrecy to sexually active adolescents [4], a finding that partly agrees with the present study. One plausible explanation is that some adolescent males with HCBs had observable attributes, e.g. a box of snuff in his pocket, reminding the GP of explaining professional secrecy, whereas poor mental health may be easier to hide. Also, it indicates that GPs presume that adolescent males with HCBs need confidentiality more than their peers. However, the present study emphasizes that regardless of HCBs, almost all adolescent males want confidentiality.

#### Asking sensitive questions

Nearly half of the adolescent males were comfortable asking sensitive questions; a perception highly influenced by experienced confidentiality. Although confidentiality is well known to facilitate discussions of sensitive topics brought up by the health-care provider [3–5,9], this is, to the best of our knowledge, the first study demonstrating that confidentiality makes adolescent males more comfortable raising their own concerns. By revealing their unmet health needs, adolescent males are more likely to receive the interventions that they need.

Adolescent males engaged in HCBs were more comfortable asking about sex, which can be interpreted as sexually active adolescent males being more at ease discussing sex than their peers. Notably, adolescent males without close friends or supportive adults other than parents were less comfortable discussing sensitive questions than their peers, whereas they valued confidentiality as much as the other respondents. This may indicate a vulnerable group that needs the GP’s attention. To find out how to make these adolescent males more comfortable asking sensitive questions, future, preferably qualitative, studies are needed.

### Meaning of the study

Confidentiality matters regardless of poor mental health or HCBs. The present study adds the perspective of adolescent males, illuminating that *private time* and *secrecy explained* are both needed for them to be comfortable sharing their own health concerns, and consequently to receive appropriate care. As to the GPs role, our results suggest that developing a habit of offering private time and explaining professional secrecy would be beneficial. Considering the heavy workload that many GPs face [39], this might be perceived as an unrealistic task. One approach that might fit the bill, however, is a standardized split-visit model [1,40]. The encounter starts by explaining the meaning and the limits of confidentiality (*secrecy explained*), followed by the *private time*, which includes sensitive parts of history taking and examination. Finally, the GP and the adolescent male summarize the assessment and proposed actions to the parents. As the present study highlights the importance of confidentiality for adolescent males to raise their own worries, we also suggest that GPs encourage such questions during the private time.
